# The Genomic Landscape of Crossover Interference in the Desert Tree *Populus euphratica*

**DOI:** 10.3389/fgene.2019.00440

**Published:** 2019-05-15

**Authors:** Ping Wang, Libo Jiang, Meixia Ye, Xuli Zhu, Rongling Wu

**Affiliations:** ^1^Center for Computational Biology, College of Biological Sciences and Biotechnology, Beijing Forestry University, Beijing, China; ^2^Center for Statistical Genetics, The Pennsylvania State University, Hershey, PA, United States

**Keywords:** euphrates poplar, genetic interference, mapping population, meiotic crossover, four-point analysis

## Abstract

Crossover (CO) interference is a universal phenomenon by which the occurrence of one CO event inhibits the simultaneous occurrence of other COs along a chromosome. Because of its critical role in the evolution of genome structure and organization, the cytological and molecular mechanisms underlying CO interference have been extensively investigated. However, the genome-wide distribution of CO interference and its interplay with sex-, stress-, and age-induced differentiation remain poorly understood. Multi-point linkage analysis has proven to be a powerful tool for landscaping CO interference, especially within species for which CO mutants are rarely available. We implemented four-point linkage analysis to landscape a detailed picture of how CO interference is distributed through the entire genome of *Populus euphratica*, the only forest tree that can survive and grow in saline desert. We identified an extensive occurrence of CO interference, and found that its strength depends on the length of chromosomes and the genomic locations within the chromosome. We detected high-order CO interference, possibly suggesting a highly complex mechanism crucial for *P. euphratica* to grow, reproduce, and evolve in its harsh environment.

## Introduction

Crossovers (COs) are recombination events involving a reciprocal exchange of genetic material. During meiotic prophase, COs are essential for the accurate segregation of homologous chromosomes (Hillers, 2004). In most organisms, the abundance and distribution of COs is highly regulated by universal mechanisms, referred to as CO interference or genetic interference. The fact that the presence of a CO interferes with the occurrence of other COs within the same chromosome has been confirmed. Due to such interferences, chiasmata are more evenly placed along chromosomes than previously expected (Hillers, 2004; Hultén, 2011). Moreover, CO interference is ubiquitous in eukaryotes and plays a crucial role in their evolution. However, our understanding of CO interference mechanisms and their distribution in biota remains very limited.

Sturtevant and Muller constructed a Drosophila genetic map and found that COs were more evenly spaced than would be expected from random placement (Lam et al., 2005). CO interference is widespread in most eukaryotes and can confer selectivity advantages. The extent of CO interference decreases with genetic distance between COs; however, given the same distance, it is stronger on the same chromosomal arm than on different arms (Berchowitz and Copenhaver, 2010). The variability of CO interference within a specific chromosome region is affected by the overall size and structure of the chromosome (Hillers, 2004), and CO interferences are regulated by the anti-recombinase RTEL-1 protein in *Caenorhabditis elegant* (Youds et al., 2010). A reduction in CO interference can result from a lack of DNA-damage-response-kinase Tel1/ATM (Anderson et al., 2015). Links between CO interferences and sex differences (Jan et al., 2007; Szatkiewicz et al., 2013), stress-induced adaptation (Yant et al., 2013; Aggarwal et al., 2015), and aging (Campbell et al., 2015; Wang Z. et al., 2016) have been discovered, highlighting the multifaceted role of COs in mediating biological processes. As an evolutionary phenotype, CO interference varies with biotic and abiotic environmental parameters, such as sex, age, and stress. For example, in mice and cattle, interference is stronger in females than in males (Szatkiewicz et al., 2013; Wang Z. et al., 2016). However, the opposite is found in humans, where interference is stronger in males than in females, although this pattern varies by chromosome (Campbell et al., 2015).

Many methods have been used to study the mechanisms of CO interference, including the count-location model, the gamma model, and multi-point linkage analysis. Initially, CO interference was genetically defined and characterized by cytology, the location of protein complexes, and chromosomal CO events. Recent studies have explored the mechanistic basis of CO interference using cytogenetics and molecular methods, whereas more traditional interference studies use the coefficient of coincidence (CoC) between two disjoint intervals on a genetic map. The CoC is defined as the ratio of the observed frequency to the expected frequency, and represents all possible intervals of gametes with double CO for each pair (Waterworth, 2000). Traditional models of interference suggest that the occurrence of a CO produces signals or substances that prevent additional CO events and then spreads along the chromosome at a similar distance on both sides (Housworth and Stahl, 2003). The polymerization model states that early recombination events are distributed independently with each other and then have the same chance of initiating bidirectional aggregation events per unit of time (King and Mortimer, 1990).

More recently, many model and non-model systems have been developed to characterize the phenomenon of CO interference. CO interference has been investigated mainly by tracking DNA markers on a single chromosome of parents during a specific period under electron fluorescence microscopy. The gamma model has recently received attention and suggests that the shape parameter of the gamma distribution is an indicator for uniformity and an indirect indicator for interference (Lam et al., 2005). The mechanical stress model assumes that each CO event releases a specific distance of pressure along the chromosome to prevent the presence of nearby COs (Wang et al., 2015). At present, multi-point linkage analysis has been proven to be more advantageous in genetic distance estimation and gene ordering, and it is equipped with a strong ability to discern and quantify CO interferences.

Despite numerous theoretical and empirical studies, our understanding of how interference is distributed across genomes remains unclear (Housworth and Stahl, 2003). This can be attributed to a number of reasons. First, traditional genetic screens for mutations affecting interference require numerous meiotic progenies to include meiotic COs in multiple intervals along a chromosome (Berchowitz and Copenhaver, 2010). Second, most of the mutations that modify interference affect chromosomal proteins, which not only mediate interference but also play a role in CO formation (Joshi et al., 2009). Thus, genetic strategies that abolish mutation interference also reduce or eliminate CO events. Third, many mutants differ in their frequency of occurrence of CO in different loci and environments (Getz et al., 2008). Therefore, combining multi-point analysis and cytology tools, which are used widely for locating and sequencing genes, can increase the ability to detect interference (Broman and Weber, 2000). The multi-analytic statistical model, which is based on the linkage analysis method of genetic maps, can describe CO interference that take place not only between two adjacent chromosome intervals, but also in multiple consecutive intervals. Additionally, multi-point analysis provides a quantitative method to estimate CO interference (Zickler and Kleckner, 2016). In particular, by assessing the chromosomal distribution of CO interference, multi-point analysis can activate the use of linkage mapping as a routine genetic tool to investigate further dimensions of genomic structure and organization (Lu et al., 2004).

*Populus euphratica* is the only arbor species in arid-semiarid regions and plays an important role in maintaining the ecological balance in desert regions. The goals of this study were to identify the distribution of CO interference in *P. euphratica* at a whole-genome scale using multi-point analysis based on the full-sib family of *P. euphratica* and to study the relationship between the overall CO interference strength and length of the chromosome, as well as the region of the chromosome. Due to the impact of climate change and anthropogenic activities, the area of *P. euphratica* in northwest China has declined sharply and its ecological security and agricultural production are facing severe challenges (Qiu et al., 2011). By using four-point linkage analysis to analyze the CO interference of *P. euphratica*, we can describe its distribution within the genome in detail, which will provide a theoretical basis for the follow-up forest genetic research and molecular marker-assisted breeding. It is of great significance to understand the genetic diversity and evolutionary history of *P. euphratica* and to find their core germplasm resources.

## Materials and Methods

### Plant Material and Genetic Linkage Map

One male and one female *P. euphratica* individual were randomly selected along the Tarim River in the Korla region of Xinjiang, China. The individuals were located 31 km from one another, ensuring a large genetic difference between them. Male and female flowering branches from the individuals were planted in an artificial climate chamber at Beijing Forestry University. After cultivation was completed, a series of experimental treatments, including dehydration, thinning, and freezing with liquid nitrogen, were performed on the selected materials. Finally, the F_1_ progeny of 408 individuals were obtained. DNA was extracted using the TIANGEN plant genomic DNA extraction kit (Beijing, China). The quality of all samples was assessed and RAD technology was used for high-throughput DNA sequencing (Conesa et al., 2005). The genetic map of *P. euphratica* was constructed from the resultant sequence data.

### Multi-Point Linkage Analysis

A four-point analysis was developed so that four consecutive markers could be analyzed simultaneously (Wang J. et al., 2016). It beyond three-point analysis, can characterize crossover interference that takes place not only between two adjacent chromosomal intervals, but also over multiple successive intervals (We call the interference occurred in multiple marker intervals of more than three markers as high dimensional CO interference). We used the CoC to describe the ratio of the observed number of double recombinants to this expected number. As we have known, the recombination events occurring between different marker intervals are not independent. Thus, the extent to which this coefficient corresponds to the strength of CO interference.

In the full-sib family of *P. euphratica*, two heterozygous F_1_ individuals, ABCD/abcd and ABCD/abcd, were crossed to produce a segregated F_2_ population. Each F_1_ parent produced 16 gametes, divided into eight types (Table 1). The frequencies of the gamete types are represented by g000,…, g111, where the subscripts represent the number of COs between a particular pair of tags. Based on the genetic map of *P. euphratica*, we grouped single-nucleotide polymorphism markers on 19 linkage groups with four markers in every group. The genotype frequencies of the gamete types were calculated by counting the number of genotypes within the 408 individuals of each group. The four consecutive markers (i.e., A-B-C-D) had six possible recombination moieties. From these gamete-type frequencies, we expressed the recombination fractions of each marker pair, denoted by *r*_AB_, *r*_BC_, *r*_CD_, *r*_AC_, *r*_BD_, and *r*_AD_, as follows:

(1) $$\begin{array}{lll} r_{AB} & = & g_{111}+g_{110}+g_{101}+g_{100}\\ r_{BC} & = & g_{111}+g_{110}+g_{011}+g_{010}\\ r_{CD} & = & g_{111}+g_{101}+g_{011}+g_{001}\\ r_{AC} & = & g_{101}+g_{100}+g_{011}+g_{010}\\ r_{BD} & = & g_{110}+g_{010}+g_{101}+g_{001}\\ r_{AD} & = & g_{111}+g_{010}+g_{100}+g_{001} \end{array}$$

Denote the coefficients of coincidence (a measure of crossover interference) between double marker intervals A-B and B-C, double marker intervals B-C and C-D, double marker intervals A-B and C-D, and triple marker intervals A-B, B-C, and C-D by *C*_1_, *C*_2_, *C*_3_, and *C*_4_, respectively (Sun et al., 2017). Wang J. et al. (2016) formulated the relationship between different recombination fractions based on the CoC and derived a process to estimate and test each coefficient, as follows:

(2) $$\begin{array}{lll} C_{4} & = & \frac{g_{111}}{r_{AB}r_{BC}r_{CD}}\\ C_{1} & = & \frac{g_{111}+g_{110}}{r_{AB}r_{BC}}\\ C_{2} & = & \frac{g_{111}+g_{011}}{r_{BC}r_{CD}}\\ C_{3} & = & \frac{g_{111}+g_{101}}{r_{AB}r_{CD}} \end{array}$$

providing a method to characterize the genomic distribution of CO interference along the chromosome.

**Table 1:** Gamete types and their frequencies at four ordered markers, A-B-C-D.

**No**.		**1**	**2**	**3**	**4**	**5**	**6**	**7**	**8**
**Gamete type**		**ABCD/abcd**	**ABCd/abcD**	**Abcd/abCD**	**ABcD/abCd**	**Abcd/aBCD**	**AbcD/aBCd**	**AbCD/aBcd**	**AbCd/aBcD**
Number of crossovers	A-B	0	0	0	0	1	1	1	1
	B-C	0	0	1	1	0	0	1	1
	C-D	0	1	0	1	0	1	0	1
Gamete type frequency		g000	g001	g010	g011	g100	g101	g110	g111

For an F_2_ offspring family of *P. euphratica*, two F_1_ progenies crossed to produce 136 diploids, divided into 81 identifiable genotypes. This situation differs from the backcross population, which is more complex and requires the Expectation Maximization algorithm to be implemented (Dempster et al., 1977). Table 2 provides the frequencies of these 81 genotypes, as well as the corresponding numbers. The frequencies of heterozygous genotypes are a mix of products of gamete-type frequencies (Wang J. et al., 2016). Subsequently, the *P. euphratica* data were analyzed by multi-point analysis to obtain the CoC values representing the CO interference strength. If the CoC value is 0, it indicates that interference is absent.

**Table 2:** Four-marker genotype observations and expected frequencies composed of gamete-type frequencies produced by each parent in a full-sib population.

**Genotype**	**g000**	**g001**	**g010**	**g011**	**g100**	**g101**	**g110**	**g111**	**Frequency**	**Observation**
AABBCCDD	2	0	0	0	0	0	0	0	g_000_^2^	n_2222_
AABBCCDd	1	1	0	0	0	0	0	0	2g_000_g_001_	n_2221_
AABBCCdd	0	2	0	0	0	0	0	0	g_001_^2^	n_2220_
AABBCcDD	1	0	0	1	0	0	0	0	2g_000_g_011_	n_2212_
AABBCcDd	^ϕ^_3_	1-^ϕ^_3_	^ϕ^_3_	1-^ϕ^_3_	0	0	0	0	2(g_000_g_010_+g_001_g_011_)	n_2211_
AABBCcdd	0	1	1	0	0	0	0	0	2g_001_g_010_	n_2210_
AABBccDD	0	0	0	2	0	0	0	0	g_011_^2^	n_2202_
AABBccDd	0	0	1	1	0	0	0	0	2g_011_g_010_	n_2201_
AABBccdd	0	0	2	0	0	0	0	0	g_010_^2^	n_2200_
AABbCCDD	1	0	0	0	0	0	1	0	2g_000_g_110_	n_2122_
AABbCCDd	^ϕ^_10_	1-^ϕ^_10_	0	0	0	0	1-^ϕ^_10_	^ϕ^_10_	2(g_000_g_111_+g_001_g_110_)	n_2121_
AABbCCdd	0	1	0	0	0	0	0	1	2g_001_g_111_	n_2120_
AABbCcDD	^ϕ^_7_	0	0	1-^ϕ^_7_	0	^ϕ^_7_	1-^ϕ^_7_	0	2(g_000_g_101_+g_011_g_110_)	n_2112_
AABbCcDd	^ϕ^_6_	^ϕ^_16_	^ϕ^_23_	^ϕ^_27_	^ϕ^_6_	^ϕ^_16_	^ϕ^_23_	^ϕ^_27_	2(g_000_g_100_+g_001_g_101_+g_010_g_110_+g_011_g_111_)	n_2111_
AABbCcdd	0	^ϕ^_15_	1-^ϕ^_15_	0	^ϕ^_15_	0	0	1-^ϕ^_15_	2(g_001_g_100_+g_010_g_111_)	n_2110_
AABbccDD	0	0	0	1	0	1	0	0	2g_011_g_101_	n_2102_
AABbccDd	0	0	^ϕ^_22_	1-^ϕ^_22_	1-^ϕ^_22_	^ϕ^_22_	0	0	2(g_010_g_101_+g_011_g_100_)	n_2101_
AABbccdd	0	0	1	0	1	0	0	0	2g_010_g_100_	n_2100_
AAbbCCDD	0	0	0	0	0	0	2	0	g_110_^2^	n_2022_
AAbbCCDd	0	0	0	0	0	0	1	1	2g_110_g_111_	n_2021_
AAbbCCdd	0	0	0	0	0	0	0	2	g_111_^2^	n_2020_
AAbbCcDD	0	0	0	0	0	1	1	0	2g_011_g_101_	n_2012_
AAbbCcDd	0	0	0	0	^ϕ^_30_	1-^ϕ^_30_	^ϕ^_30_	1-^ϕ^_30_	2(g_110_g_100_+g_111_g_101_)	n_2011_
AAbbCcdd	0	0	0	0	1	0	0	1	2g_111_g_100_	n_2010_
AAbbccDD	0	0	0	0	0	2	0	0	g_101_^2^	n_2002_
AAbbccDd	0	0	0	0	1	1	0	0	2g_101_g_100_	n_2001_
AAbbccdd	0	0	0	0	2	0	0	0	g_100_^2^	n_2000_
AaBBCCDD	1	0	0	0	1	0	0	0	2g_000_g_100_	n_1222_
AaBBCCDd	^ϕ^_8_	1-^ϕ^_8_	0	1-^ϕ^_8_	0	^ϕ^_8_	0	0	2(g_000_g_101_+g_001_g_011_)	n_1221_
AaBBCCdd	0	1	0	0	0	1	0	0	2g_001_g_101_	n_1220_
AaBBCcDD	^ϕ^_11_	0	0	1-^ϕ^_11_	1-^ϕ^_11_	0	0	^ϕ^_11_	2(g_000_g_111_+g_011_g_100_)	n_1212_
AaBBCcDd	^ϕ^_9_	^ϕ^_18_	^ϕ^_21_	^ϕ^_26_	^ϕ^_21_	^ϕ^_26_	^ϕ^_9_	^ϕ^_18_	2(g_000_g_110_+g_001_g_111_+g_010_g_100_+g_011_g_101_)	n_1211_
AaBBCcdd	0	^ϕ^_17_	1-^ϕ^_17_	0	0	1-^ϕ^_17_	^ϕ^_17_	0	2(g_001_g_110_+g_010_g_101_)	n_1210_
AaBBccDD	0	0	0	1	0	0	0	1	2(g_010_g_111_+g_011_g_110_)	n_1202_
AaBBccDd	0	0	^ϕ^_24_	1-^ϕ^_24_	0	0	1-^ϕ^_24_	^ϕ^_24_	2(g_010_g_111_+g_011_g_110_)	n_1201_
AaBBccdd	0	0	1	0	0	0	1	0	2g_010_g_110_	n_1200_
AaBbCCDD	^ϕ^_4_	0	^ϕ^_4_	0	1-^ϕ^_4_	0	1-^ϕ^_4_	0	2(g_000_g_010_+g_110_g_100_)	n_1122_
AaBbCCDd	^ϕ^_5_	^ϕ^_13_	^ϕ^_13_	^ϕ^_5_	^ϕ^_31_	^ϕ^_33_	^ϕ^_33_	^ϕ^_31_	2(g_000_g_011_+g_001_g_010_+g_110_g_101_+g_111_g_100_)	n_1121_
AaBbCCdd	0	^ϕ^_14_	0	^ϕ^_14_	0	1-^ϕ^_14_	0	1-^ϕ^_14_	2(g_001_g_011_+g_111_g_101_)	n_1120_
AaBbCcDD	^ϕ^_2_	^ϕ^_2_	^ϕ^_20_	^ϕ^_20_	^ϕ^_29_	^ϕ^_29_	^ϕ^_35_	^ϕ^_35_	2(g_000_g_001_+g_011_g_010_+g_110_g_111_+g_101_g_100_)	n_1112_
AaBbCcDd	2^ϕ^_1_	2^ϕ^_12_	2^ϕ^_19_	2^ϕ^_25_	2^ϕ^_28_	2^ϕ^_32_	2^ϕ^_34_	2^ϕ^_36_	g_000_^2^+g_001_^2^+g_010_^2^+g_100_^2^+g_101_^2^+g_110_^2^+g_011_^2^+g_111_^2^)	n_1111_
AaBbCcdd	^ϕ^_2_	^ϕ^_2_	^ϕ^_20_	^ϕ^_20_	^ϕ^_29_	^ϕ^_29_	^ϕ^_35_	^ϕ^_35_	2(g_000_g_001_+g_011_g_010_+g_110_g_111_+g_101_g_100_)	n_1110_
AaBbccDD	0	^ϕ^_14_	0	^ϕ^_14_	0	1-^ϕ^_14_	0	1-^ϕ^_14_	2(g_001_g_011_+g_111_g_101_)	n_1102_
AaBbccDd	^ϕ^_5_	^ϕ^_13_	^ϕ^_13_	^ϕ^_5_	^ϕ^_31_	^ϕ^_33_	^ϕ^_33_	^ϕ^_31_	2(g_000_g_011_+g_001_g_010_+g_110_g_101_+g_111_g_100_)	n_1101_
AaBbccdd	^ϕ^_4_	0	^ϕ^_4_	0	1-^ϕ^_4_	0	1-^ϕ^_4_	0	2(g_000_g_010_+g_110_g_100_)	n_1100_
AabbCCDD	0	0	1	0	0	0	1	0	2g_010_g_110_	n_1022_
AabbCCDd	0	0	^ϕ^_24_	1-^ϕ^_24_	0	0	1-^ϕ^_24_	^ϕ^_24_	2(g_010_g_111_+g_011_g_110_)	n_1021_
AabbCCdd	0	0	0	1	0	0	0	1	2g_011_g_111_	n_1020_
AabbCcDD	0	^ϕ^_17_	1-^ϕ^_17_	0	0	1-^ϕ^_17_	^ϕ^_17_	0	2g_011_g_111_	n_1012_
AabbCcDd	^ϕ^_9_	^ϕ^_18_	^ϕ^_21_	^ϕ^_26_	^ϕ^_21_	^ϕ^_26_	^ϕ^_9_	^ϕ^_18_	2(g_000_g_110_+g_001_g_111_+g_010_g_100_+g_011_g_101_)	n_1011_
AabbCcdd	^ϕ^_11_	0	0	1-^ϕ^_11_	1-^ϕ^_11_	0	0	^ϕ^_11_	2(g_000_g_111_+g_011_g_100_)	n_1010_
AabbccDD	0	1	0	0	0	1	0	0	2g_001_g_101_	n_1002_
AabbccDd	^ϕ^_8_	1-^ϕ^_8_	0	1-^ϕ^_8_	0	^ϕ^_8_	0	0	2(g_000_g_101_+g_001_g_011_)	n_1001_
Aabbccdd	1	0	0	0	1	0	0	0	2g_000_g_100_	n_1000_
aaBBCCDD	0	0	0	0	2	0	0	0	g_100_^2^	n_0222_
aaBBCCDd	0	0	0	0	1	1	0	0	2g_101_g_100_	n_0221_
aaBBCCdd	0	0	0	0	0	2	0	0	g_101_^2^	n_0220_
aaBBCcDD	0	0	0	0	1	0	0	1	2g_111_g_100_	n_0212_
aaBBCcDd	0	0	0	0	^ϕ^_30_	1-^ϕ^_30_	^ϕ^_30_	1-^ϕ^_30_	2(g_110_g_100_+g_111_g_101_)	n_0211_
aaBBCcdd	0	0	0	0	0	1	1	0	2g_011_g_101_	n_0210_
aaBBccDD	0	0	0	0	0	0	0	2	g_111_^2^	n_0202_
aaBBccDd	0	0	0	0	0	0	1	1	2g_110_g_111_	n_0201_
aaBBccdd	0	0	0	0	0	0	2	0	g_110_^2^	n_0200_
aaBbCCDD	0	0	1	0	1	0	0	0	2g_010_g_100_	n_0122_
aaBbCCDd	0	0	^ϕ^_22_	1-^ϕ^_22_	1-^ϕ^_22_	^ϕ^_22_	0	0	2(g_010_g_101_+g_011_g_100_)	n_0121_
aaBbCCdd	0	0	0	1	0	1	0	0	2g_011_g_101_	n_0120_
aaBbCcDD	0	^ϕ^_15_	1-^ϕ^_15_	0	^ϕ^_15_	0	0	1-^ϕ^_15_	2(g_001_g_100_+g_010_g_111_)	n_0122_
aaBbCcDd	^ϕ^_6_	^ϕ^_16_	^ϕ^_23_	^ϕ^_27_	^ϕ^_6_	^ϕ^_16_	^ϕ^_23_	^ϕ^_27_	2(g_000_g_100_+g_001_g_101_+g_010_g_110_+g_011_g_111_)	n_0111_
aaBbCcdd	^ϕ^_7_	0	0	1-^ϕ^_7_	0	^ϕ^_7_	1-^ϕ^_7_	0	2(g_000_g_101_+g_011_g_110_)	n_0110_
aaBbccDD	0	1	0	0	0	0	0	1	2g_001_g_111_	n_0102_
aaBbccDd	^ϕ^_10_	1-^ϕ^_10_	0	0	0	0	1-^ϕ^_10_	^ϕ^_10_	2(g_000_g_111_+g_001_g_110_)	n_0101_
aaBbccdd	1	0	0	0	0	0	1	0	2g_000_g_110_	n_0100_
aabbCCDD	0	0	2	0	0	0	0	0	g_010_^2^	n_0022_
aabbCCDd	0	0	1	1	0	0	0	0	2g_011_g_010_	n_0021_
aabbCCdd	0	0	0	2	0	0	0	0	g_011_^2^	n_0020_
aabbCcDD	0	1	1	0	0	0	0	0	2g_001_g_010_	n_0012_
aabbCcDd	^ϕ^_3_	1-^ϕ^_3_	^ϕ^_3_	1-^ϕ^_3_	0	0	0	0	2(g_000_g_010_+g_001_g_011_)	n_0011_
aabbCcdd	1	0	0	1	0	0	0	0	2g_000_g_011_	n_0010_
aabbccDD	0	2	0	0	0	0	0	0	g_001_^2^	n_0002_
aabbccDd	1	1	0	0	0	0	0	0	2g_000_g_001_	n_0001_
aabbccdd	2	0	0	0	0	0	0	0	g_000_^2^	n_0000_

ϕ *refers to the ratio of the frequency of each gamete genotype to the corresponding genotype frequency*.

### The Relationship Between Overall High Dimensional CO Interference Strength and Chromosome Length

Differences in CO interference strength are affected by the overall size of the chromosome (Albini, 2010). Through four-point linkage analysis, we obtained the recombination rate between four marker intervals on each linkage group and the corresponding CoC. To study the relationship between chromosome length and overall high-order CO interference strength, we assumed that the length of the linkage group on the genetic map was the length of the chromosome. Next, the distribution interval of high dimensional CO interference strength on the 19 chromosomes was characterized by a boxplot displaying the maximum, minimum, median, and upper and lower quartiles of the data. Due to different structural characteristics of chromosomes, there are many factors affecting the strength of CO interference; therefore, the mean of the CO interference strength on each chromosome was calculated to account for the relationship between chromosome size and overall CO interference strength. Due to the distribution of chromosome 1 deviates more from the distribution of other chromosomes, it was determined to be an outlier and was removed from the dataset. Subsequently, chromosomes 2, 3, 4, and 6 were fitted with a linear model (blue line), and the remaining chromosomes were fitted with a trend line (red line). Through the fitting curves, the distribution of the overall high dimensional CO interference strength on different chromosomes was observed.

### Ratio Variance in High Dimensional CO Interference Strength Between Different Chromosome Regions

CO rates are closely related to chromosome region (Giraut et al., 2011), allowing for differences in CO interference strength in different regions to be explored. In this study, each chromosome was divided into three parts according to genetic distance uniformity, and the three sections were labeled NO.1, NO.2, and NO.3, respectively. The CO interference strength of each was subtracted separately. NO.1-NO.2, NO.2-NO.3, and NO.1-NO.3 indicate the difference ratio (sum of the difference value of each corresponding CO interference strength between intervals) of CO interference strength in the first (NO.1) and second (NO.2) parts, the second part and the third (NO.3) part, the first and third part, respectively. This allowed for differences in the distribution of CO interference strength between the regions of the chromosome to be seen.

To display the impact of the three regions (NO.1, NO.2, and NO.3) in the chromosome on the CO interference strength distribution, we employed δ to quantitatively evaluate the difference of the CO interference strength distribution in different sections of chromosome, which can be calculated by

(3) $$\begin{array}{l} \delta \text{=}\overset{N}{\underset{i=1}{\sum }}|p_{i}^{1}-p_{i}^{2}| \end{array}$$

where *N* is the total number of intervals of the CO interference strength value, $p_{i}^{1}$ and $p_{i}^{2}$ represent the percentage of the *i*th interval in two different chromosome regions, respectively. We further derived the range of δ:

(4) $$\begin{array}{l} 0\leq \delta \text{=}\overset{N}{\underset{i=1}{\sum }}|p_{i}^{1}-p_{i}^{2}|\leq \overset{N}{\underset{i=1}{\sum }}p_{i}^{1}+\overset{N}{\underset{i=1}{\sum }}p_{i}^{2}=2 \end{array}$$

When the CO interference strength distributions in both regions 1 and 2 were the same, δ was equal to 0, whereas δ reached the maximum of 2 when there was no overlapping region between the CO interference strength distributions of two regions. In all other cases, δ is larger than 0 and smaller than 2. δ reflects the difference of two different CO interference strength distributions.

## Results

In this study, we first used a four-point linkage analysis model to quantitatively analyze the CO interference on a full-sib population of *P. euphratica*. The genetic map contained 8,305 markers on 19 linkage groups. The total genetic distance was 4574.89 cM for the entire genetic map, among which the shortest linkage group was linkage group 19 (LG19) with a genetic distance of 130.26 cM and the longest linkage group was LG1 with a distance of 530.03 cM. The average distance of markers on each individual linkage group was 0.40–0.66 cM (Zhang et al., 2017).

**Figure 1:**
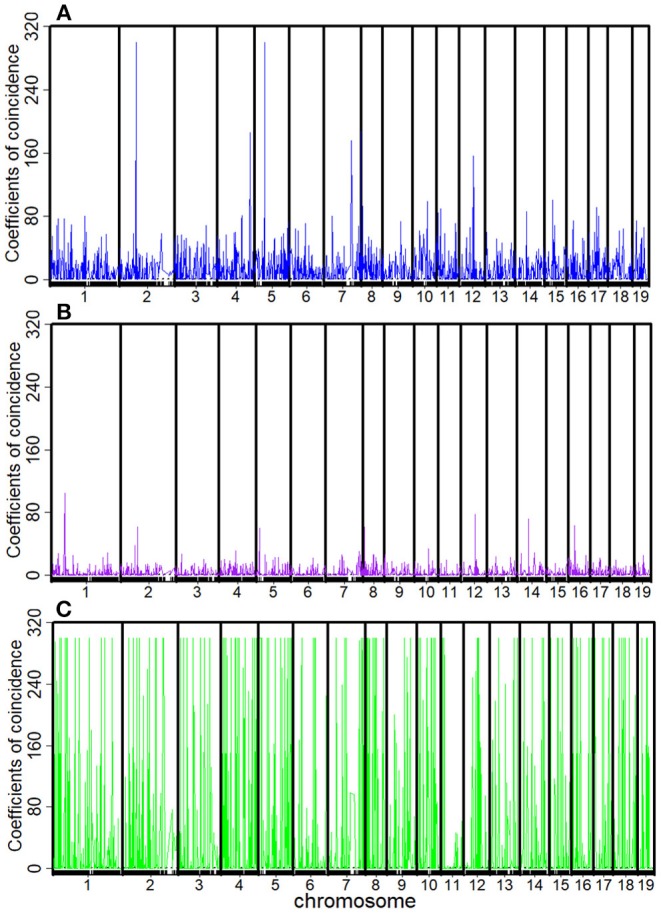
Distribution of crossover interference within the *Populus euphratica* genome, composed of 19 chromosomes, estimated from a full-sib family of two different cultivars. **(A)** Crossover interference between two adjacent marker intervals (*C*_1_ and *C*_2_); **(B)** crossover interference between two non-adjacent marker intervals (*C*_3_); **(C)** high-dimensional crossover interference over three successive marker intervals (*C*_4_).

The recombination rates *r*_AB_, *r*_BC_, *r*_CD_, *r*_AC_, *r*_BD_, and *r*_AD_ and the corresponding *C*_1_, *C*_2_, *C*_3_, and *C*_4_ between every four consecutive markers were obtained by four-point linkage analysis (Table 3). According to the CoC (Table 3) and the genetic distance of each linkage group, we determined the CO interference between two adjacent intervals, the CO interference of one interval apart, and the high dimensional CO interference of triple marker intervals. CO interference is ubiquitous within a genome, exhibiting COs between two adjacent marker intervals distributed throughout the genome and varied with the length of the chromosome (Figure 1A), making the distribution of COs across each linkage group more even. However, the distribution of interference between two non-adjacent marker intervals occasionally occurs at lower frequencies and lower intensities than the adjacent intervals (Figure 1B). Interestingly, high dimensional CO interference was highly distributed across the 19 linkage groups and had a wide distribution within the genome (Figure 1C). By comparison, high dimensional CO interference with high-density distribution existed on linkage group 4 (LG4) and linkage group 5 (LG5), whereas the high-dimensional CO interference distribution density of linkage group 11 (LG11) was lower.

**Table 3:** Recombination rates corresponding to the first two groups in each linkage group and the coefficient of coincidence (CoC) of crossover interference strength.

**r_**AB**_**	**r_**BC**_**	**r_**CD**_**	**r_**AC**_**	**r_**BD**_**	**r_**AD**_**	**C1**	**C2**	**C3**	**C4**	**lg**	**Marker 1**	**Marker 2**	**Marker 3**	**Marker 4**
0.50	0.02	0.50	0.50	0.50	0.03	1.00	1.00	1.91	0.00	1	nn_np_92921	lm_ll_11315	lm_ll_4392	nn_np_8171
0.02	0.03	0.50	0.04	0.50	0.50	13.46	1.00	1.00	13.46	1	lm_ll_12452	lm_ll_7926	lm_ll_9277	nn_np_8634
0.02	0.04	0.02	0.04	0.04	0.04	11.53	15.37	7.69	177.29	2	lm_ll_10351	lm_ll_4075	lm_ll_11480	lm_ll_9220
0.02	0.03	0.01	0.02	0.03	0.02	21.12	20.52	11.15	98.29	2	lm_ll_9058	hk_hk_3051	hk_hk_1972	hk_hk_2298
0.04	0.03	0.03	0.05	0.02	0.04	9.03	19.87	0.00	0.00	3	lm_ll_11125	lm_ll_8568	lm_ll_8093	lm_ll_4728
0.05	0.02	0.03	0.05	0.03	0.04	10.94	16.30	8.30	71.58	3	lm_ll_11556	hk_hk_2534	hk_hk_3271	hk_hk_1110
0.01	0.01	0.05	0.01	0.05	0.05	54.89	6.12	3.50	0.01	4	hk_hk_2770	hk_hk_3044	hk_hk_1608	nn_np_5712
0.50	0.50	0.50	0.01	0.02	0.50	1.97	1.96	1.93	3.86	4	lm_ll_10753	nn_np_12108	lm_ll_8376	nn_np_12562
0.03	0.04	0.09	0.03	0.09	0.08	17.70	5.68	0.52	14.20	5	hk_hk_3094	hk_hk_2811	hk_hk_616	hk_hk_2812
0.02	0.02	0.04	0.03	0.05	0.06	11.40	7.24	3.95	96.10	5	hk_hk_2689	hk_hk_3110	hk_hk_3313	hk_hk_1097
0.01	0.01	0.01	0.02	0.01	0.01	21.62	48.28	0.00	0.00	6	hk_hk_1495	hk_hk_3053	hk_hk_2055	hk_hk_2954
0.01	0.01	0.02	0.01	0.01	0.02	44.45	37.22	9.80	873.87	6	hk_hk_2493	hk_hk_3029	hk_hk_3009	hk_hk_1331
0.05	0.02	0.06	0.05	0.06	0.01	9.32	8.49	12.63	0.00	7	hk_hk_2701	hk_hk_2144	hk_hk_2510	hk_hk_1471
0.03	0.08	0.02	0.08	0.09	0.10	6.61	2.55	4.50	57.32	7	hk_hk_2148	hk_hk_1178	hk_hk_2076	hk_hk_2322
0.03	0.03	0.03	0.03	0.02	0.02	15.60	23.40	0.00	0.00	8	lm_ll_12636	lm_ll_7930	lm_ll_10007	lm_ll_9837
0.02	0.02	0.02	0.01	0.03	0.02	46.77	6.82	0.00	0.01	8	lm_ll_3885	lm_ll_11525	lm_ll_9742	lm_ll_12405
0.02	0.01	0.02	0.02	0.02	0.03	31.41	20.19	0.00	0.02	9	nn_np_8717	nn_np_10414	nn_np_12617	nn_np_9786
0.02	0.04	0.01	0.03	0.05	0.03	20.74	2.42	22.34	0.00	9	hk_hk_3317	hk_hk_1551	hk_hk_2994	nn_np_11331
0.04	0.50	0.01	0.50	0.50	0.50	1.00	1.00	0.00	0.00	10	lm_ll_7856	lm_ll_5397	nn_np_8256	nn_np_7630
0.50	0.01	0.50	0.50	0.50	0.02	1.00	1.00	1.92	0.00	10	nn_np_11692	lm_ll_12780	lm_ll_6166	nn_np_5449
0.50	0.02	0.50	0.50	0.50	0.04	1.00	1.00	1.91	0.77	11	hk_hk_2928	hk_hk_3329	hk_hk_2833	hk_hk_2138
0.49	0.04	0.07	0.51	0.11	0.46	0.64	0.67	1.68	0.00	11	hk_hk_871	hk_hk_1707	hk_hk_1946	nn_np_7373
0.02	0.09	0.03	0.08	0.11	0.11	10.25	3.16	11.69	123.68	12	hk_hk_2068	hk_hk_2300	hk_hk_2504	hk_hk_682
0.12	0.01	0.01	0.11	0.01	0.11	6.08	32.58	2.77	0.00	12	hk_hk_2654	hk_hk_1675	hk_hk_2586	hk_hk_253
0.03	0.03	0.50	0.04	0.50	0.50	11.43	1.00	1.00	11.43	13	nn_np_5159	nn_np_5866	nn_np_4850	lm_ll_1263
0.50	0.50	0.50	0.01	0.01	0.50	1.98	1.98	1.96	3.93	13	nn_np_11032	lm_ll_9285	nn_np_10951	lm_ll_12427
0.03	0.06	0.02	0.07	0.05	0.06	5.50	13.21	0.00	0.00	14	lm_ll_4169	lm_ll_8617	lm_ll_11626	lm_ll_10819
0.06	0.01	0.07	0.07	0.07	0.13	0.00	7.58	0.00	0.00	14	lm_ll_12378	lm_ll_12011	lm_ll_6388	lm_ll_9230
0.50	0.03	0.04	0.50	0.02	0.50	1.00	20.20	1.00	20.20	15	nn_np_12714	lm_ll_5117	lm_ll_9900	lm_ll_10761
0.01	0.03	0.02	0.02	0.02	0.01	28.20	24.42	6.55	0.03	15	nn_np_10876	nn_np_6544	nn_np_10233	hk_hk_1031
0.04	0.03	0.50	0.03	0.50	0.50	16.13	1.00	1.00	16.13	16	lm_ll_7848	lm_ll_12916	lm_ll_12998	nn_np_10507
0.50	0.00	0.03	0.50	0.04	0.50	1.00	0.00	1.00	0.00	16	lm_ll_9352	nn_np_3807	nn_np_8544	nn_np_6630
0.50	0.50	0.01	0.01	0.50	0.02	1.98	1.00	1.00	1.60	17	nn_np_11597	lm_ll_8106	nn_np_7356	nn_np_10281
0.04	0.02	0.04	0.06	0.04	0.05	3.91	14.56	4.25	0.00	17	hk_hk_2057	lm_ll_12267	lm_ll_8687	lm_ll_2545
0.03	0.02	0.04	0.04	0.03	0.03	9.25	16.94	11.26	38.84	18	hk_hk_3194	hk_hk_2315	hk_hk_2540	hk_hk_2773
0.03	0.02	0.01	0.03	0.03	0.04	16.50	9.08	0.00	0.01	18	nn_np_10997	nn_np_3830	nn_np_10927	nn_np_12341
0.07	0.03	0.50	0.10	0.50	0.50	0.00	1.00	1.00	0.00	19	lm_ll_2856	lm_ll_12917	lm_ll_9631	nn_np_10203
0.50	0.50	0.50	0.02	0.02	0.50	1.96	1.97	1.92	3.85	19	nn_np_10340	lm_ll_10921	nn_np_9928	lm_ll_2421

**Figure 2:**
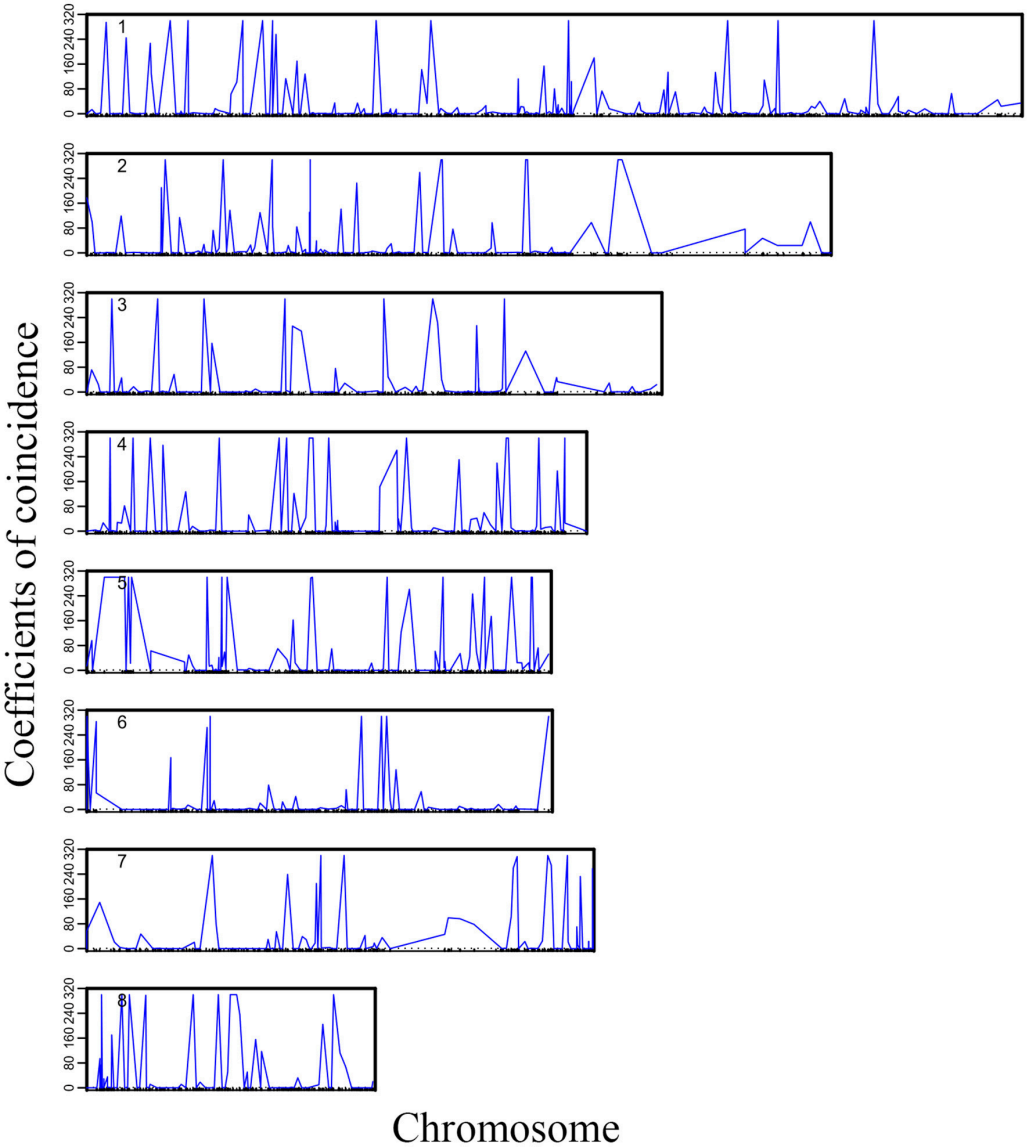
Landscape of crossover interference along eight chromosomes within the *Populus euphratica* genome.

We plotted the first eight high-dimensional CO interference in the 19 linkage groups to visualize the distribution of high-dimensional CO interference on the eight linkage groups more directly (Figure 2). Although the chromosome length varied, higher-dimensional CO interferences were evenly distributed within each chromosome and the amplitudes were larger and denser than the other two genetic disturbances. Additionally, the location information of the markers where CO interference occurred could be seen (Figure 2). There was an obvious correlation between the density of high-dimensional CO interference and chromosome length, with different chromosome lengths resulting in different distributions of high-dimensional CO interference.

**Figure 3:**
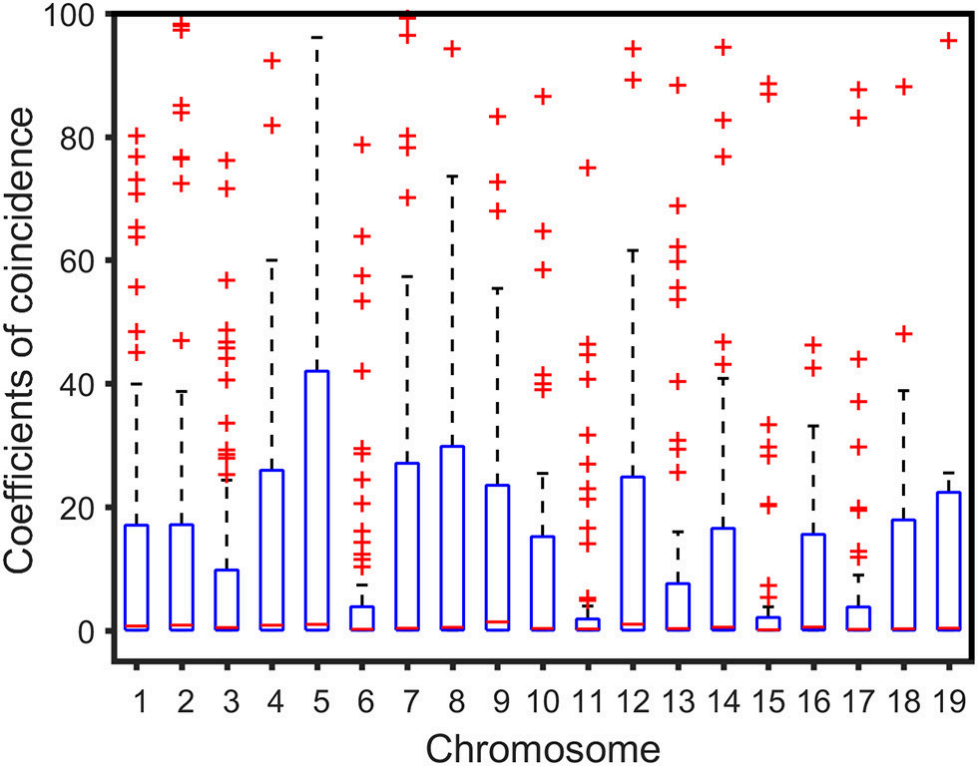
Boxplot of crossover interference over the *Populus euphratica* genome composed of 19 chromosomes.

**Figure 4:**
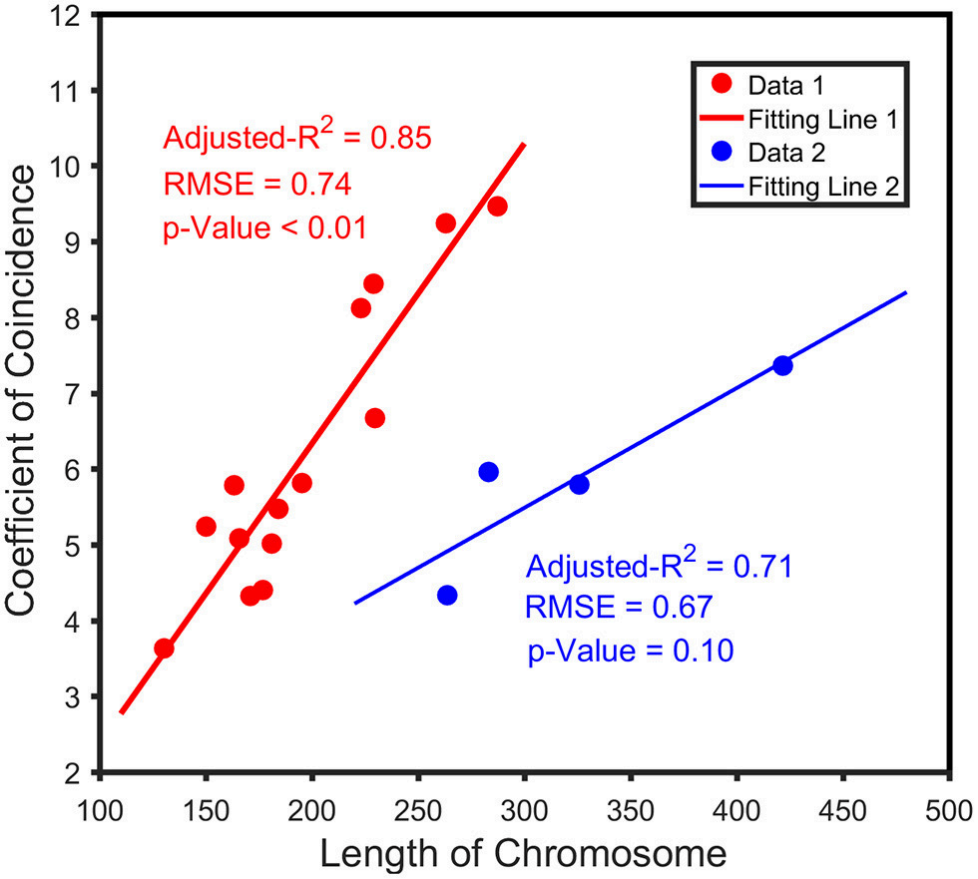
Relationship between the length of the chromatid and the coefficient of coincidence (CoC). The blue line indicates the trend between crossover interference and the chromosome length of chromosomes 2, 3, 4, and 6; the red line indicates the trend of genetic interference of the remaining chromosomes with chromosome length.

We analyzed the correlation between the genetic distance of chromosomes and overall high-dimensional CO interference strength. The median of the overall CO interference strength was concentrated between 0 and 1, and the interquartile range (IQR) was variable and dependent on chromosome length. The IQR of chromosome 5 was the longest, reaching 41.63 cM; the IQR of chromosome 11 was the shortest, about 1.74 cM; the other 17 chromosomes were similar to chromosome 1, which was about 16.94 cM (Figure 3). In other words, the overall strength of CO interference was related to the genetic distance of the chromosome (Figure 4). Chromosomes 2, 3, 4, and 6 were locally linearly fitted (blue line) with an adjusted *R*^2^ of 0.71. Simultaneously, the other chromosomes were fitted (red line) with an adjusted *R*^2^ of 0.85 (Figure 4). Although the two fitted curves had different slopes, they both increased with the length of the chromosome. These results suggest that the correlation between the genetic distance of chromosomes and the overall high-dimensional CO interference strength was significant.

**Figure 5:**
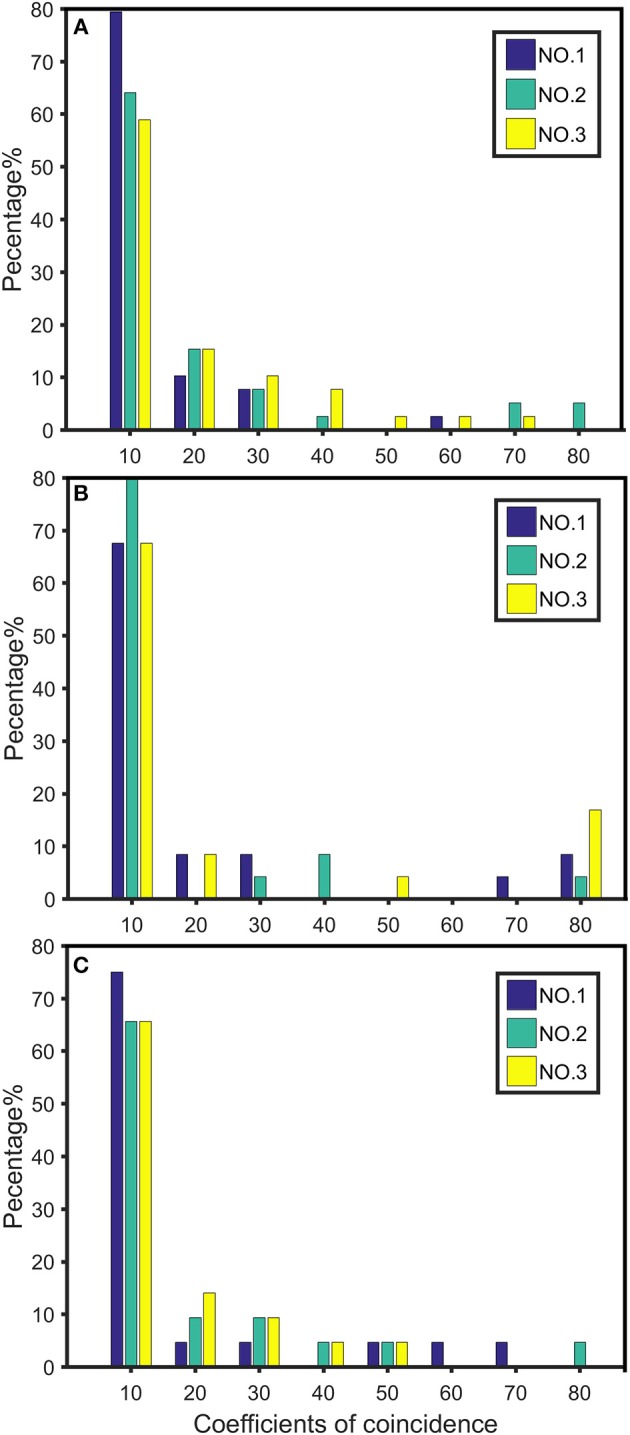
Distribution of crossover interference strength on chromosomes 1 **(A)**, 2 **(B)**, and 3 **(C)**. NO.1 represents the distribution strength of the first third of the chromosome length; NO.2 represents the distribution strength of the middle third of the chromosome; NO.3 indicates the distribution strength of the last third of the chromosome.

We plotted the first three of the 19 chromosomes to visualize the distribution of high dimensional CO interference on different chromosome parts (NO.1, NO.2, and NO.3) (Figure 5). The CO interference strength of each chromosome part differed in terms of intensity interval. For example, on chromosome 1, there was no CO interference in the first part (interval of 60–80 cM), whereas chromosome 2 exhibited CO interference. Therefore, different intervals along the chromosome contained different strengths and distributions of CO interference.

**Figure 6:**
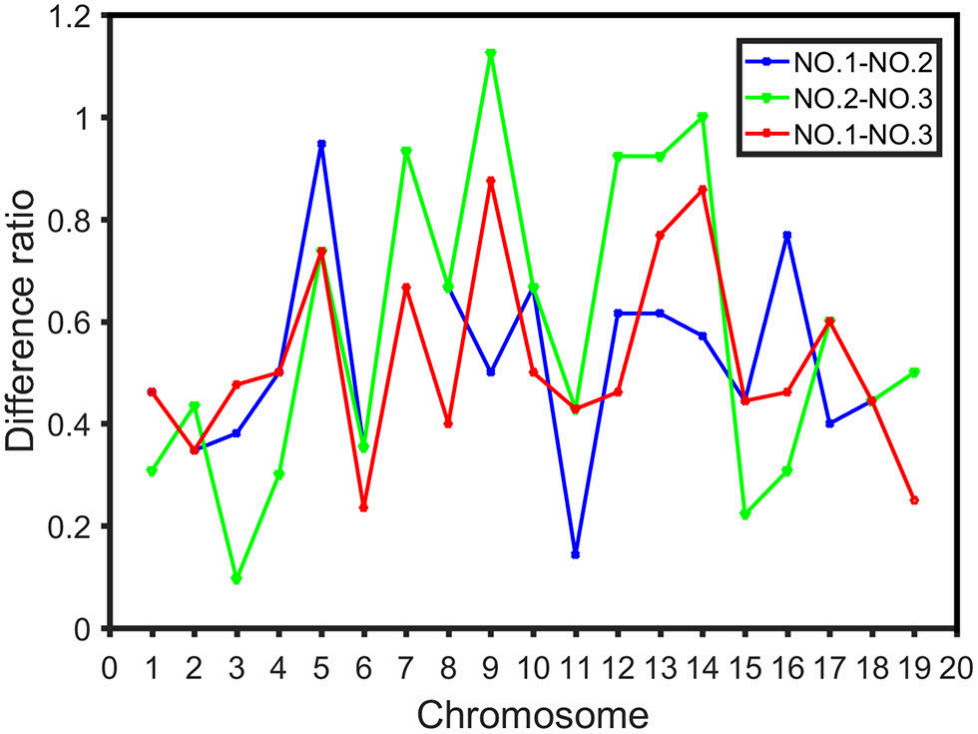
Difference ratio in the distribution of CO interference between the three parts of the chromosome, where the blue line represents the difference ratio between the first part (NO.1) and the middle part (NO.2), the green line represents the difference ratio between the middle part (NO.2) and the third part (NO.3), and the red line indicates the difference ratio between the first part (NO.1) and the third part (NO.3).

The difference ratio was used to compare the differences among the three intervals on each chromosome and study the distribution of high dimensional CO interference strength in different regions of the chromosome. The difference ratios of NO.1-NO.2, NO.2-NO.3, and NO.1-NO.3 in each chromosome were 0.1429-0.9474, 0.0952-1.1250, and 0.2353-0.8750, respectively (Figure 6). Moreover, fluctuations of CO interference strength between the first region and the third region were small, whereas the CO interference strength between the second region and the third region fluctuated greatly (Figure 6). The high dimensional CO interference strength between the middle region and both side regions on the chromosome was very different. Thus, the overall strength of high dimensional CO interference was not only related to the length of the chromosome, but also varied among chromosome regions.

## Discussion

The phenomenon of CO interference has been observed in most organisms. Within eukaryotes, interference may be quite long. For example, in the nematode *C. elegans*, interference can span a fusion chromosome of 50 Mb (Lian et al., 2008). The results of this study provide strong evidence for the existence of high-order CO interference. We assessed CO interference in the full-sib family of *P. euphratica* by mapping the distributions of CO interferences in different dimensions along 19 chromosomes. We observed that high-dimensional CO interference existed to varying degrees on all 19 chromosomes, and found that these high-dimensional interferences were even stronger than one- or two-dimensional CO interferences. The discovery of CO interference in the full-sib family of *P. euphratica* and the relationship between the strength of the overall CO interference and the chromosome structure can not only help identify and quantify CO interference in the entire genome, but also has the potential to impact further inference on the genome structure, organization, and evolution of *P. euphratica* populations.

We correlated the genetic length of the chromosome with the strength of the overall high-dimensional CO interference, and found that the mean of CO interference strength on each chromosome had a linear relationship with the genetic length of the chromosome. CO rates and chromosome lengths were previously found to be relevant in other eukaryotic species, including humans, mice, *Arabidopsis*, and zebrafish (Kleckner et al., 2003). In addition, CO interference affects the CO rate and is affected by the length of the chromosome. In some species, such as yeast, dogs, mice, and pigeons, small chromosomes often have a higher CO density (Froenicke et al., 2002; Basheva et al., 2008; Mancera et al., 2008). Surprisingly, the CO interference strength in this study increased with chromosome length, with longer chromosomes containing a higher CO interference density and a correspondingly smaller CO density. This finding has far-reaching implications on biological evolution. Due to the existence of CO interference, the occurrence of CO events is regulated accordingly (Broman et al., 2002). The length of chromosomes indirectly affects the total strength of heritage interference, thereby affecting genetic diversity and having important implications for evolution.

According to previous studies, the occurrence of CO events is closely related to the center and terminal regions on chromosomes (Chelysheva et al., 2007). Meanwhile, CO interference has variable intensities and distributions in different regions of the chromosome. Moreover, CO interference can have different regulatory effects on a CO event in the corresponding region and exerts subtle influences on biological inheritance and evolution. We further studied the distribution and difference of CO interference between different regions on the chromosome, finding that the distribution of CO interference strength differed among regions. By defining the range of difference ratios, we found a difference in CO interference strength among chromosome regions. Studies of *Arabidopsis* chromosomes have shown that CO rates correlate with different genomic features associated with chromosome structure, such as the GC content and CpG ratio. Therefore, the differences in CO interference are also clearly related to these factors.

In this study, we used multi-point analysis methods to measure CO interference in the full-sib family of *P. euphratica*, extending from traditional linkage analysis to analyze multiple markers simultaneously. Previous studies have demonstrated that this method is a powerful tool for identifying and estimating CO interference (Wang J. et al., 2016). Accurate estimates of high-dimensional CO interference have significant implications in genomic research (Weeks et al., 1994). First, previous studies of interference in experimental organisms generally only involved adjacent interval groups, whereas multi-point analysis can not only accurately estimate the recombination rate between two adjacent markers, but also between multiple marker intervals and provide additional information about genomic structure and organization. Second, using this method, the strength and distribution of CO interferences between adjacent intervals along a chromosome can be estimated and the results can be used to study the relationship with the structure of the chromosome.

An increasing number of studies have investigated the phenomenon of CO interference. It has been found that CO interference is highly related to many evolutionary and developmental processes, such as gender differences, heterogeneity, senescence, and stress tolerance. The distribution of recombination achieved by CO interference can be determined by genetic background, gender, and many environmental factors, such as temperature and age. However, most genetic mapping studies have not considered CO interference. Regardless, multi-point analysis using genetic mapping has been used to estimate the degree of correlation between CO interference and evolution, and can capture this important phenomenon without extra cost. Similarly, Aggarwal et al. (2015) used multi-point analysis to determine the rules of recombinant frequency and CO interference in fruit flies that were targeted by dry, hypoxia, or high-oxygen tolerance. Here, we have expanded the research on CO interference, allowing for future studies to explore the molecular mechanism of CO in the *P. euphratica* genome through combination of multi-point analysis with cytology, clarify the development and evolution of COs, and investigate whether specific genes regulate CO interference.

## Author Contributions

PW performed data analysis. PW, MY, and XZ interpreted the result. RW and LJ conceived of the idea and designed the model. PW and LJ wrote the manuscript.

### Conflict of Interest Statement

The authors declare that the research was conducted in the absence of any commercial or financial relationships that could be construed as a potential conflict of interest.
